# Supplementary material for: Enhancing Health and Empowerment: Assessing the Satisfaction of Underprivileged Rural Women Participating in a Functional Literacy Education Program in Kailali District, Nepal
Source: Healthcare (Basel). 2024 May 27;12(11):1099. doi: 10.3390/healthcare12111099 (PMC11172237; doi:10.3390/healthcare12111099)
Supplement: Supplementary file 1 [file healthcare-12-01099-s001.zip › healthcare-2938680-supplementary.pdf]

Supplementary Figure S1

Scale: ALL VARIABLES

Case Processing Summary

		N	%
Cases	Valid	141	100.0
	Excluded[a]	0	.0
	Total	141	100.0

a. Listwise deletion based on all variables in the procedure.

Reliability Statistics

Cronbach's Alpha	Cronbach's Alpha Based on Standardized Items	N of Items
.726	.821	16
